# Involving young people and parents in decision-making for hypodontia

**DOI:** 10.1038/s41415-023-6328-1

**Published:** 2023-10-13

**Authors:** Sophy Barber, Adam Jones, Veena Abigale Patel, Martin P. Ashley

**Affiliations:** 41415382693001 Clinical Lecturer, University of Leeds, UK; Honorary Consultant Orthodontist, Mid Yorkshire NHS Hospitals Trust, UK; 41415382693002 Academic Clinical Fellow in Oral Surgery, Leeds Dental Institute, UK; 41415382693003 Academic Clinical Fellow in Orthodontics, Leeds Dental Institute, UK; 41415382693004 Consultant and Honorary Professor in Restorative Dentistry and Oral Health, University Dental Hospital of Manchester, UK

## Abstract

Involving young people and their parents in decisions about their health care is ethically and professionally the right thing to do. Good decision-making relies on informed, value-based deliberation. Providing the right treatment for people with hypodontia is complex, both technically, in terms of the range of options available, and from a communication perspective. Treatment decisions faced by young people with hypodontia can have lifelong implications and the weight of this is felt both by the patient, who may have limited experience of dental treatment and decision-making, and their parents, who act as advocates. It is important that clinicians understand how they can best share the available evidence and their expertise in a way that can be understood and applied. Clinicians also have an important role in facilitating young people to recognise and communicate their own values, expectations, and ultimately, preferences for treatment. This paper outlines the challenges of navigating information sharing and engaging in shared decision-making specific to hypodontia. A scoping review of the literature by the authors was conducted to identify evidence-based advice for discussing uncertainties, risks and increasing engagement in decision-making. This may be useful to both primary and secondary care practitioners involved in decision-making with people with hypodontia.

## Key points

- Choosing the 'best' treatment for a young person with hypodontia is challenging due to the variation in clinical presentation and treatment options, differences in patients' understanding, goals and expectations, and variability in clinical services.
- Shared decision-making (SDM) is a joint process in which healthcare professionals and patients work together to make decisions about their care. The decision is reached by taking into account the clinician's clinical knowledge, expertise, the available evidence base and the patient's preferences, beliefs and values.
- Establishing how the decision-maker wishes to be involved, how much information they want, and how they prefer information to be delivered is essential to ensure communication is person-centred and supports deliberative decision-making.
- Communication of risks, benefits and consequences, particularly when using numerical information, can be challenging. Discussions may be aided by the use of plain language, summary tables to present all of the risks and benefits of each option side by side, presenting information in several formats, use of visual aids and using absolute rather than relative risks.

## Introduction

**Fig. 1:**
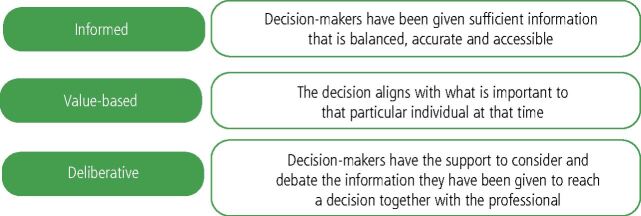
Principals of clinical decision-making

Good decision-making in healthcare is informed, value-based and deliberative (Fig. 1). The majority of clinical decision-makingoccurs during consultations and for people with hypodontia, this commonly involves the dental team, the person with hypodontia, and anyone else who is important to the decision, such as family members. All parties bring a different and important perspective to the consultation and this can pose a number of challenges. Clinicians understand and consider clinical parameters (for example, the endodontic risk of needing to prepare teeth for a conventional bridge versus a resin-bonded bridge), whereas patients consider personal parameters (for example, how many appointments, will it hurt, how many years until treatment is finished, days off school and work, distance to travel to provider).

Variable levels of knowledge and understanding about the diagnosis, the decision to be made and the treatment options can inhibit effective patient-professional communication. A lack of time may also limit the opportunity to understand what is important and deliberate about the different options and their relative risks and benefits. The biases and assumptions of all involved may lead to ineffective information sharing and deliberation, for example, believing that a specific risk or outcome is not important to a particular individual, or assuming that someone may not wish to discuss a treatment option because it is only available privately. Patients and families may have already been given information by their general dental practitioner that may have influenced their beliefs about which treatment is 'best' treatment. Furthermore, dental professionals will have different perspectives on what the appropriate treatment options are; this variation in practice may be reduced by working together in clinical networks, undertaking peer review of cases, national audits and benchmarking.

The NHS advocates shared decision-making (SDM) for all non-emergency decisions about treatment where more than one option exists, including the option of no treatment.^1^ SDM is a joint process in which healthcare professionals and patients work together to make decisions about their care. The decision is reached by taking into account the clinician's clinical knowledge and expertise, the available evidence-base and the patient's preferences, beliefs and values.^2^ SDM differs from, but is related to, the process of informed consent, which is a minimum legal requirement for clinicians to share information through its higher level of patient-centredness in which patients are invited to engage in a two-way information-sharing process and actively consider all options and what is important to them.^3^ There are a number of different definitions and proposed models of SDM; however, all share the overarching aim to support individuals to make the decision that is right for them at that time. The key components that have been suggested to come together to support SDM are summarised in Table 1.^4^^,^^5^^,^^6^^,^^7^^,^^8^

**Table 1:** Key components of SDM

Access to information before the decision-making consultation	Comprehensive and unbiased information should ideally be available to the patient and family in advance to enable effective information exchange during the consultation.
Confirmation of the patient's existing knowledge	Patients are unlikely to be aware of all of the treatment options and their risks and benefits, so establishing current knowledge will help to tailor the conversation to where knowledge is lacking. Checking knowledge can also help to identify any misconceptions that could lead to maladaptive behaviours when seeking treatment.
Promoting choice awareness	Patients at this stage may have an idea which option they would prefer based on their own values and what they deem to be important - known as intitial preferences. It is important to ensure the patient is aware there is a choice to be made and that they have enough information to develop informed preferences.
Establishing the preferred level of participation	Determining whether the patient prefers that they make the decision alone, the clinician makes the decision, or that they make a joint decision together.
Information sharing	Information regarding all viable treatment options is given, as well as the differences between options, risks, benefits, advantages and disadvantages.
Eliciting preferences and values	It is important to find out what the patient's preferences and values are. For example, by asking: 'some of the pros and cons of the options that I have been through will matter to you more than others. What are your thoughts about them?'.
Emphasise the role of uncertainty	In medicine and dentistry, there may be inadequate evidence for certain treatments, and if this is the case, patients should be made aware of this. For example, 'there is no evidence to say that one method of treatment is better than the other. Therefore, the choice will be based around what you find most important'.
Supporting deliberation	Deliberation is the process by which the patient becomes aware of choice and has time to understand their options. The patient should be supported in considering what matters most to them. Value clarification methods, decision aids and decision counselling can be used to help identify and understand patient preferences.
Reaching a decision	If the patient has expressed a decision, list the options again to assess understanding and check that there have not been any misconceptions. If the patient is not ready to make a decision, it is important to ascertain why. They may wish for more information or simply time to go away and think about the options. A further review (not necessarily face-to-face) should be offered to facilitate this.
Reassessing decisions throughout treatment	Patient values and preferences may change throughout treatment. These should be explored so changes can be made to the treatment plan to accommodate the patient's wishes.

Ethically, involving patients in decisions about their own treatment is the right thing to do. The General Dental Council's* Standards for the dental team* highlights that patients expect to have their preferences taken into account and their values respected, while practitioners have a compulsory duty to discuss all options in an accessible way, consider individuals' preferences and values, and support deliberation.^9^ SDM has been misrepresented as a method of increasing efficiency within healthcare institutions, reducing costs and reducing litigation but there is insufficient evidence to show that this is the case. The motivations for clinicians to engage in SDM should be that of undertaking good clinical practice in the form of patient-centred care, where patients are told that their informed preferences should be the basis of reaching a decision that is right for them. SDM should not be used to limit resources and save money.^10^There is also evidence to show that patients would like to be more involved in decisions about their care than they currently are.^11^^,^^12^ The suggested benefits of SDM include improved patient knowledge, involvement and satisfaction; reduction in complaints; increased adherence to treatment and self-care; improved health outcomes; and more effective use of resources.^1^^,^^5^^,^^13^ Importantly, SDM may benefit disadvantaged groups more than groups with higher literacy, education and socioeconomic status and hence, be a mechanism for reducing health inequality.^14^This is interesting considering the need for patients to understand, balance options and engage in discussions with the clinical team; an important question may be whether disadvantaged groups benefit as much as is needed through SDM to reach parity with non-disadvantaged groups.

## Navigating the challenges of decision-making in hypodontia

### Knowledge and understanding

Hypodontia poses some specific challenges for decision-making, related to the condition itself, the treatment options and characteristics of the decision-makers. Diagnosis commonly occurs in early adolescence when one or more permanent teeth fail to erupt.^15^ At this time, young people may have limited experience of both dental treatment and making healthcare decisions, so parents often have an important role as an advocate for their child;^16^ however, parents themselves report that they do not always fully understand the treatment options and that they feel a huge responsibility to choose the 'right' treatment for their child.

### Accessible information

Written information in accessible formats is recommended to support verbal discussions with clinicians and to facilitate deliberation outside of a clinical environment, but a review of information resources for hypodontia found a lack of comprehensive online or written patient information has previously been identified.^17^ Since this review, a website specifically about hypodontia has been developed and tested with evidence of increased patient knowledge through engagement with the website.^18^ However, to the authors' knowledge, this website is not currently in widespread use. Relevant, accessible, high-quality, evidence-based resources about hypodontia that are co-produced between patients and clinicians are essential for supporting patients to make informed decisions.

### Expectations and goals

The impact of hypodontia varies depending on the individual and perhaps surprisingly, impact does not necessarily correlate with the number of missing teeth.^19^^,^^20^^,^^21^ Functional and oral-health-related quality of life (OHRQoL) impact are reported for hypodontia across a number of studies^19^^,^^20^^,^^21^^,^^22^^,^^23^^,^^24^^,^^25^^,^^26^^,^^27^^,^^28^and this impact is a key influence on patients' expectations, motivation for treatment and their goals. A hypodontia-specific quality of life measure has been shown to be more discriminative for measuring OHRQoL,^24^ while a condition-specific tool that examines expectations about treatments^29^has also been reported. There could be great value in adapting these tools for routine clinical use to explore patient concerns and expectations for treatment before or during consultations.

### Establishing preferred level of engagement in decision-making

The 2021 National Institute for Health and Care Excellence (NICE) *Shared decision making* guideline^2^ provides clear recommendations for engaging patients and family members before, during and after discussions. This includes asking about their preferred level of involvement, who should be included in discussions and any support needs. Many young people with hypodontia and their parents may already feel able to engage in decision-making to their preferred level but for others it may be necessary to explain what being involved means and establish their preferred role. While there are no specific tools that are advocated for establishing preferred roles, a number of general campaigns across healthcare have attempted to raise patient and family awareness of how they can be more engaged in choices about treatment, such as Advancing Quality Alliance's 'Ask Three Questions' (adapted from work by Shepherd *et al.)* ^30^ and Health Improvement Scotland's 'What matters to you?' campaign.

### Sharing information and supporting deliberation

The suitability of different treatment options depends on the individual's clinical presentation and management in the permanent dentition often involves interdisciplinary treatment from different dental specialities.^15^^,^^31^ The Manchester hypodontia team uses the 6W Principles to enhance multidisciplinary work, which involves asking:

- Why does the patient want treatment?
- HoW do we propose to provide this treatment?
- Who will be responsible for each stage and who will have overall responsibility for managing this patient?
- Where will each stage of treatment be provided?
- When will the treatment commence?; When will the important stages occur?; When is treatment likely to be completed?
- What clinical outcomes are we expecting?

These questions are clinician-focused but can be easily adapted to support discussion with patients and their families to ensure all important information has been discussed.

### Personalised approach

**Fig. 2:**
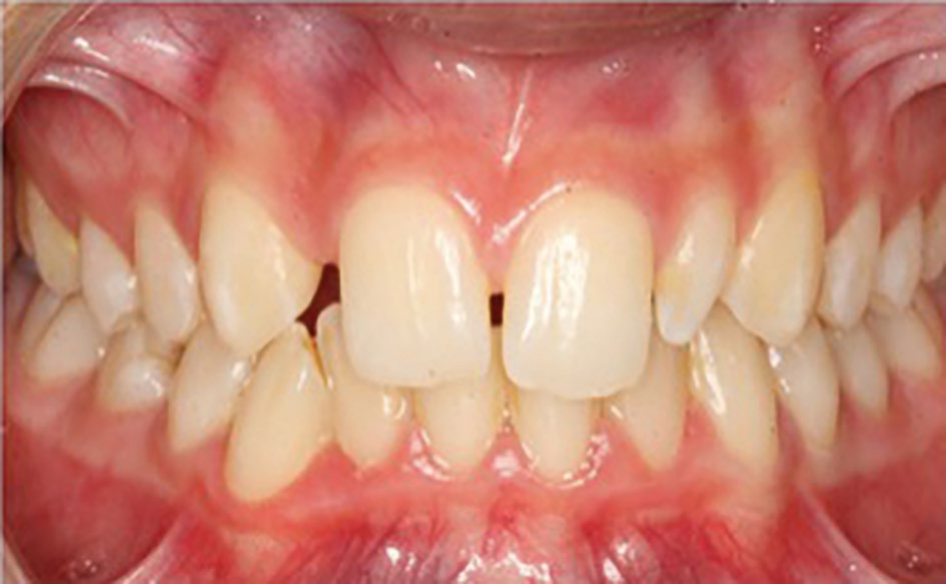
A young person with developmentally absent 12 and microdont 22. This case demonstrates the complexities of decision-making for even mild hypodontia

In many cases, there is no single 'best' treatment, so patient goals and preferences are fundamental to selecting treatment and a personalised approach is required. This can be demonstrated by considering the case, shown in Figure 2, in which a decision is required about treatment for a developmentally absent maxillary right lateral incisor (12) and microdont maxillary left lateral incisor (22). Given the ability to effectively reposition, restore and replace teeth using contemporary orthodontic and restorative dentistry techniques, there are multiple potential treatment options, including:

1. No treatment and accept the dentition. This may be chosen where the patient has no concerns or all forms of treatment are contra-indicated
2. Accept tooth position and undertake restorative camouflage. This is unlikely to provide an optimal aesthetic outcome but may be chosen by a patient who is unwilling or unable to undergo orthodontic treatment
3. Orthodontic space closure:
  - With extraction of the microdont 22 and bilateral space closure with canine substitution and camouflage
  - Maintenance of the 22 microdont and unilateral space closure with restorative camouflage of the microdont and substitute canine. This may present challenges for achieving symmetry
4. Orthodontic space opening:
  - Maintenance and restorative camouflage of the microdont 22 and tooth replacement of 12. This may again present challenges for achieving symmetry
  - With extraction of microdont 22 and bilateral tooth replacement of the maxillary lateral incisors.

There is no evidence to suggest that one treatment is superior to another and it is therefore important to establish what is important to the patient in terms of the complexity, length and risks of treatment, aesthetic and functional outcome, and potential long-term maintenance requirements. The is no 'right' decision and the most appropriate treatment will depend on that individual's preferences at that time.

### Establishing patient preferences

Preference elicitation is central to SDM but often, patients may require support to explore the different options, identify what is important to them, and reach an informed preference. A preference elicitation study measuring young people's and parents' choices for different hypothetical treatments for hypodontia found respondents made different choices depending on whether their priority was optimal aesthetics, improving function, or avoiding risk and discomfort.^32^ This highlights the importance of establishing what is important to the individual in terms of outcome from treatment and also their willingness and ability to accept different types of treatment. Patients may need to be supported to identify exactly what they want to get from treatment and the trade-offs that they are willing to make to achieve this, including level of risk and willingness to make long-term lifestyle changes, such as dietary changes, improved oral hygiene and restrictions on hobbies, such as contact sports.

There is growing interest in decision support tools, such as patient decision aids (PDAs), which are tools that help people make informed choices by explicitly identifying the decision to be made, providing information about the options and clarifying personal values and preferences.^33^ PDAs have been shown to have a beneficial effect on knowledge, value identification, risk perception and active participation in decision-making.^34^ Currently, there are no PDAs or other decision tools to support patient-dentist decision-making about dental treatment for hypodontia.

### Discussing uncertainty and risk

One of the important aspects of SDM highlighted in the NICE *Shared decision making* guideline^2^ relates to communication of risks, benefits and consequences, particularly when using numerical information. Healthcare decisions almost always involve some degree of diagnostic, prognostic or optimal-treatment uncertainty. Uncertainty arises from inadequacies in scientific knowledge, such as missing or inconsistent evidence, or difficulty translating population-level research to individual circumstances; this is known as epistemic uncertainty. There can also be problems with access to and application of existing scientific knowledge, which are compounded when the evidence is complex, poorly understood, or if the decision-maker does not have the ability to use the available information in their decision process.^35^ The evidence base for different hypodontia treatment strategies is limited by the lack of consistency in the choice of outcomes and measures, with few patient-centred outcomes.^36^

Aleatoric (random) uncertainties relate to variability over which we have no control, such as differences in disease instances across a population. In hypodontia, there may be variation in the significance of specific risks for individuals and how these relate to patient treatment preferences. Aleatoric uncertainty also describes future developments that may affect the outcome of treatment in the long-term, but which cannot be predicted.^37^ When making treatment decisions with young people with hypodontia, there are uncertainties around how their dental needs may change as they age, for example, in response to changing lifestyle, or expectations, or in response to development of dental disease, such as caries or periodontal conditions. It is important to address these uncertainties during the decision-making phase, while recognising that it might be impossible to appreciate their full significance.

Risks are an unavoidable aspect of healthcare and discussion of the risks involved with any form of dental treatment, including no treatment, is central to making informed decisions. To be in a position to make the necessary trade-offs between risk and benefit, decision-makers must fully understand the information being presented to them. Health literacy and numeracy are important considerations when presenting risk information in a format patients will find useful for decision-making. Health literacy is the ability to understand the words clinicians use to discuss health-related decisions but this is commonly low among the general public.^38^ Numeracy is important when using numbers or statistics to make decisions^39^ but it is should be appreciated that numeracy of the public is generally lower than that of clinicians.^40^ Delivery of information should account for this. Asking questions about how an individual prefers to receive information is a good starting point in this respect. Box 1 summarises evidence-based suggestions for optimising conveyance of written and numerical information.^35^

It is common for clinicians to feel uncomfortable when discussing risk and uncertainty with patients and there is a misconception among professionals that admitting uncertainty will have a negative impact on patient relationships or undermine confidence. Research actually suggests that patients highly value these discussions^41^^,^^42^and that clinician-led expressions of uncertainty improve engagement and satisfaction, while strengthening the clinician-patient relationship.^42^ It is also important to recognise that not discussing uncertainty can result in a false sense of certainty and that informed decision-making is not possible without explicit discussion of the uncertainty that complicates many decisions.^43^

There are some general principles which can be applied to improve patient understanding of risk and uncertainty.^35^ The research about how best to discuss uncertainty is more limited but it may help to begin by directly addressing how much information decision-makers want and how they prefer this to be delivered. This allows tailoring of the conversation to meet the individual's needs and expectations. It might also be helpful to clarify whether uncertainty in the decision-making process is important to young people and their families and if so, which types of uncertainty they find most distressing, followed by an assurance that their questions will be answered as thoroughly as possible.

Box 1General principles to improve patient understanding of risk and uncertainty

- Using plain language
- Using summary tables to present all of the risks and benefits of each option side by side
- Presenting information in several formats
- Using absolute rather than relative risks and rounding off numbers to avoid false impressions of precision
- Using visual aids (such as pictographs) which can improve cognitive outcomes
- Awareness that the order risks and benefits are presented can affect perception
- Awareness that framing of information can be persuasive
- Considering presenting only the information that is most relevant to the decision being made, even at the expense of completeness.

### Identifying decisional uncertainty and revisiting decisions

For many people, the information exchange process will mean that they are able to reach a decision they are happy with, but for some, there may be a need to identify any residual decisional uncertainty. Numerous tools have been used in research to measure decisional uncertainty, most commonly the Decisional Conflict Scale^44^ which has been used in nearly 400 studies to date.^45^ A shorter screening tool, such as the four-question SURE (sure of myself; understand information; risk-benefit ratio; encouragement),^46^^,^^47^ may be more easily used within the clinical setting to identify those who have residual uncertainty and the root cause of this uncertainty. For example, SURE identifies whether the individual is unsure about the risks and benefits of each option, unsure which are important to them, or that they need additional support or advice.

In milder forms of hypodontia, it may be possible to provide a definitive treatment, which means there is no need to revisit treatment decisions, such as space closure for one or two missing teeth; however, in more severe presentations, treatment may continue throughout life as primary teeth are lost or new options for tooth replacement, such as dental implants, become available. This long-term treatment burden may have a significant impact on the individual with hypodontia and their family^48^and there may be a change in understanding, expectations and goals over time.^27^ Whenever treatment options change, it is important that patients are given the opportunity to re-discuss their concerns, goals, preferences and choices.

### Evaluating decision-making practice

As with other clinical processes, there is value in evaluating current decision-making practice to identify good practice and where improvements could be made; however, this can be challenging because consideration needs to be given to what should be measured, from whose perspective and using which tool. Evaluation could examine the whole decision-making process, completion of key steps in the process, satisfaction with the process, the decision itself, satisfaction with the decision, or the health outcome from the decision. Each of these could be examined from the perspective of the patient and family, the professional, an objective observer, or a combination of these. There are a number of tools that have been used to evaluate decision-making,^49^ but these have predominantly been used in research and not all have been validated for use with young people or child-parent dyads. These issues with selecting a valid measure have been experienced by hospital trusts that wish to include evidence of SDM as a key performance indicator. While there is no clear best measure to use, if decision-making is to be evaluated, it is important that the tool selected aligns with the purpose of the evaluation.

## Conclusion

Involving young people and parents in decisions about hypodontia is essential but it presents particular challenges. There are a range of complex potential treatment options, which are highly preference-sensitive and have long-term implications. Young people and their parents often begin to navigate these decisions with limited knowledge or experience of dentistry and healthcare decisions. Parental advocacy is important but parents may have different values or expectations about treatment and parents report feeling a huge responsibility when making decisions on behalf of their child. Clinicians can facilitate informed, deliberative and value-based decision-making by encouraging young people to identify and articulate their own expectations and preferences. Considering how clinicians can best convey their knowledge and expertise or present the relevant research is important.
